# A novel technique to characterize procoagulant platelet formation and evaluate platelet procoagulant tendency in cats by flow cytometry

**DOI:** 10.3389/fvets.2024.1480756

**Published:** 2024-12-16

**Authors:** Meg Shaverdian, Nghi Nguyen, Ronald H. L. Li

**Affiliations:** ^1^Department of Surgical and Radiological Sciences, School of Veterinary Medicine, University of California, Davis, Davis, CA, United States; ^2^Department of Clinical Sciences, College of Veterinary Medicine, North Carolina State University, Raleigh, NC, United States

**Keywords:** cardiogenic arterial thromboembolism, hypertrophic cardiomyopathy, mitochondrial membrane potential, phosphatidylserine, platelet activation

## Abstract

**Introduction:**

Cardiogenic arterial thromboembolism (CATE) is a life-threatening complication of hypertrophic cardiomyopathy (HCM) with a high mortality rate. As the primary responders in hemostasis, platelets play a crucial role in the progression of CATE. Procoagulant platelets are a subpopulation of activated platelets that facilitate thrombin generation to strengthen thrombus structure. Since their discovery, procoagulant platelets have gained a considerable amount of attention due to their potential role in thromboembolic complications. Uncovering the key phenotypic markers and agonists underlying the procoagulant response in feline platelets may provide diagnostic and therapeutic insights in the treatment of CATE. However, species differences in platelet physiology and the sensitive nature of feline platelets pose some significant challenges in studying procoagulant platelets in cats.

**Objectives:**

To first devise a flow cytometric method to sequentially assess procoagulant platelet markers and to identify agonists that could induce procoagulant platelet phenotypes. Furthermore, a novel scoring system was established to evaluate the procoagulant tendency of platelets in cats.

**Methods:**

Platelets were isolated from eight healthy cats and activated by thrombin in the presence or absence of collagen (COL) and convulxin (CVX). The following markers were measured sequentially by flow cytometry: (1) loss of mitochondrial membrane potential (ΔΨm), (2) phosphatidylserine (PS) externalization, and (3) P-selectin upregulation.

**Results:**

Thrombin in the presence of CVX significantly reduced ΔΨm and induced P-selectin upregulation (*p* = 0.0078, *p* = 0.0128, respectively). In addition, thrombin-treated platelets in the presence of COL and CVX augmented PS externalization significantly (*p* = 0.043, *p* = 0.0172, respectively). Of the agonists tested, thrombin and CVX resulted in the highest procoagulant tendency score with 75% cats reaching a score ≥2/3. The number of cats with a procoagulant tendency score of 2 or 3 for thrombin and thrombin + COL was 4/8 (50%) and 5/8 (62.5%), respectively.

**Discussion:**

Sequential analysis of procoagulant markers via flow cytometry may be an effective technique for studying procoagulant platelets in cats.

## Introduction

Cardiogenic arterial thromboembolism (CATE) is a devastating complication of cardiomyopathies in cats with a high mortality rate. Hypertrophic cardiomyopathy (HCM), which affects up to approximately 30% of older cats, is the most common cause of CATE (1–3). CATE arises when a thrombus within the left atrium or left atrial appendage embolizes to distal arteries causing impedance of blood flow, tissue ischemia, and necrosis (1, 2). Although the exact pathophysiology of CATE is not fully understood, platelets, being the main effector cells in hemostasis, play a crucial role in the pathogenesis, and advancement of this complication. Multiple studies in cats with HCM indicate that increased platelet adhesion and activation may exacerbate intracardiac thrombosis via platelet priming, shedding of platelet-derived microvesicles and upregulation of adhesion molecules like platelet-endothelial cell adhesion molecule-1 and von Willebrand factor (4–7).

The antiplatelet drug, clopidogrel, is the recommended thromboprophylaxis for primary and secondary prevention of CATE in cats with cardiomyopathies (8, 9). Despite broad usage of clopidogrel, CATE recurrence remains high, which indicates that current anti-platelet therapies are inadequate at preventing intracardiac thrombosis and CATE (10). In addition to clopidogrel resistance, another plausible cause of variable drug response is that cats with HCM may have a higher propensity to form procoagulant platelets (11–13). Although there are studies that show that ADP receptor inhibitors like clopidogrel and cangrelor can decrease procoagulant platelet formation in humans, conventional antiplatelet drugs may not target procoagulant platelets in cats given the species differences and sensitivity to agonists (13–15). Procoagulant platelets are a subpopulation of platelets that differ from aggregating platelets based on their role in hemostasis. Upon vascular injuries, aggregating platelets undergo adhesion, and activation in response to mechanical shear and nearby agonists, forming pseudopodia with high affinity integrins. This further facilitates aggregation with nearby platelets forming the majority of a thrombus core (13). As a portion of platelets within the thrombus are exposed to high concentrations of potent agonists, their persistent activation transforms them into procoagulant platelets (13). Unlike aggregating platelets, procoagulant platelets have inactive integrins, and high amounts of electronegative phospholipids like phosphatidylserine (PS) on the external leaflets of the cell membrane, which facilitates the binding of coagulation complexes to amplify thrombin generation and fibrinogen coating (13, 16).

While procoagulant platelets play a pivotal role in fortifying clot structures, numerous studies have suggested their role in thromboembolic diseases (17–20). Recent studies conducted in human beings showed a potential association between procoagulant platelets and thrombotic diseases. Increased procoagulant platelets formed by *ex vivo* stimulation with thrombin and collagen were observed in patients with cardiovascular events like cortical ischemic stroke, transient ischemic stroke and coronary artery disease (17–19). Individuals with a greater propensity for forming procoagulant platelets are also at risk of stroke recurrence (20). Procoagulant platelets, therefore, can serve as novel biomarkers in cats with HCM as they may enable veterinarians to reliably stratify cats at risk of CATE and devise appropriate thromboprophylaxis to prevent thrombosis. However, an important first step in assessing procoagulant platelets in clinical cats is to identify their distinguished cellular features since some of their phenotypes overlap with those of apoptotic platelets (21). However, the distinct species differences in platelet physiology may pose some challenges when studying procoagulant platelets in cats. Given the potential clinical implications for procoagulant platelets as a biomarker and therapeutic targets in feline CATE, a novel technique in assessing the propensity to form procoagulant platelets is needed. Herein, we described a flow cytometric method to sequentially assess procoagulant platelet markers in cats and to identify agonists that could induce procoagulant platelet phenotypes. Lastly, we devised a novel scoring system to assess the procoagulant tendency in individual cats.

## Materials and methods

### Animals

Eight cats from a colony of Maine Coon/outbred mixed domestic cats from the University of California, Davis HCM Research Laboratory were enrolled in this study. Of the 8 cats, 5 were heterozygous and 3 were wild type for the A31P variant in the myosin binding protein C gene, *MYBP3*. Cats ranged from 1 to 4 years of age. Half of the cats were intact males, and the other half were intact females. All cats received a physical examination by the corresponding author and a complete blood count using an automated hematology analyzer (HM5, Zoetis, Parsippany, NJ). All cats had transthoracic echocardiogram performed as described and none of the cats had echocardiographic evidence of HCM at the time of the study (5). This study was approved by the Institutional Animal Care and Use Committee of the University of California, Davis (protocol number: 22376). Four to 6 ml of blood, collected from either the medial saphenous or jugular vein using a 21- or 23-gauge butterfly needle, was transferred immediately to tubes containing 3.2% trisodium citrate. Each tube was gently inverted a couple of times and inspected for any possible clotting.

### Platelet rich plasma generation

Citrated blood tubes were placed in a 37°C bead bath within 15 min of blood collection. Blood tubes were incubated for 30 min to facilitate erythrocyte sedimentation before centrifugation for 5 min at 300 × g (21°C, no brakes). Platelet rich plasma (PRP) was then extracted using siliconized glass pipettes, and placed in round-bottom polypropylene tubes. Isolated PRP was then carefully examined for “swirling” to ensure that they maintained their resting discoid shape. Platelet counts of PRP were obtained using an automated hematology analyzer (HM5, Abaxis, Parsippany, NJ) and confirmed by blood smear analysis. PRP were rested for an additional 30 min at 37°C before activation.

### Procoagulant platelet formation and detection

**Figure 1:**
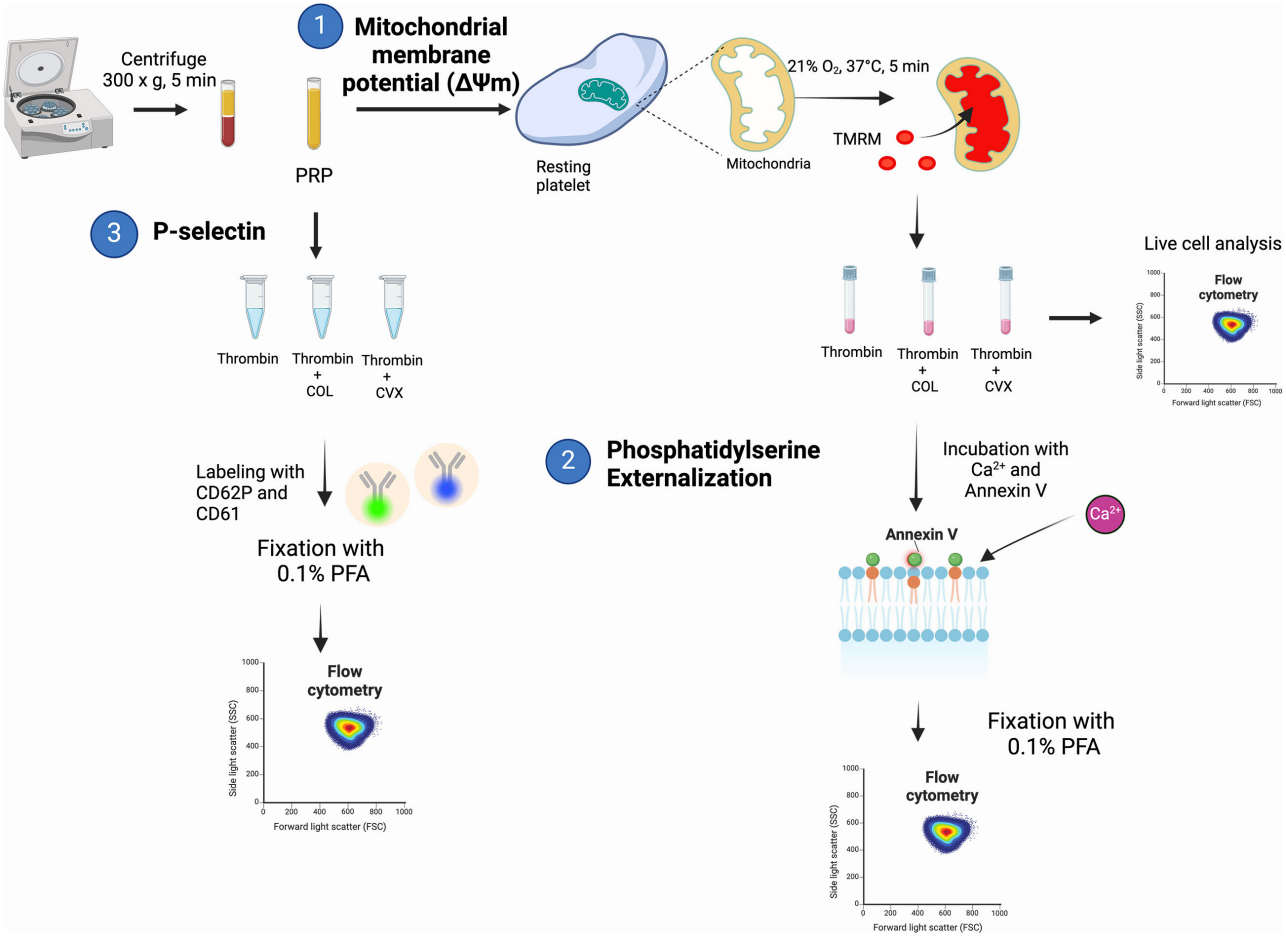
A schematic diagram summarizing the experimental procedures to evaluate procoagulant platelet markers in feline platelets. (1) Platelet rich plasma (PRP) was loaded with tetramethylrhodamine methyl ester (TMRM) to measure agonist-induced changes in mitochondrial membrane potential. (2) Samples were subsequently incubated with additional calcium and measurement of phosphatidylserine externalization with fluorophore-conjugated annexin V on flow cytometry after fixation with 0.1% paraformaldehyde (PFA). (3) Separate aliquots of PRP were assessed for P-selectin expression on flow cytometry after stimulation with thrombin, thrombin and collagen (COL) or thrombin and convulxin (CVX).

The workflow for characterizing markers of procoagulant platelet is summarized in Figure 1 with the corresponding steps described below:

#### 1) Measurement of mitochondrial membrane potential (ΔΨm) in live platelets

**Figure 2:**
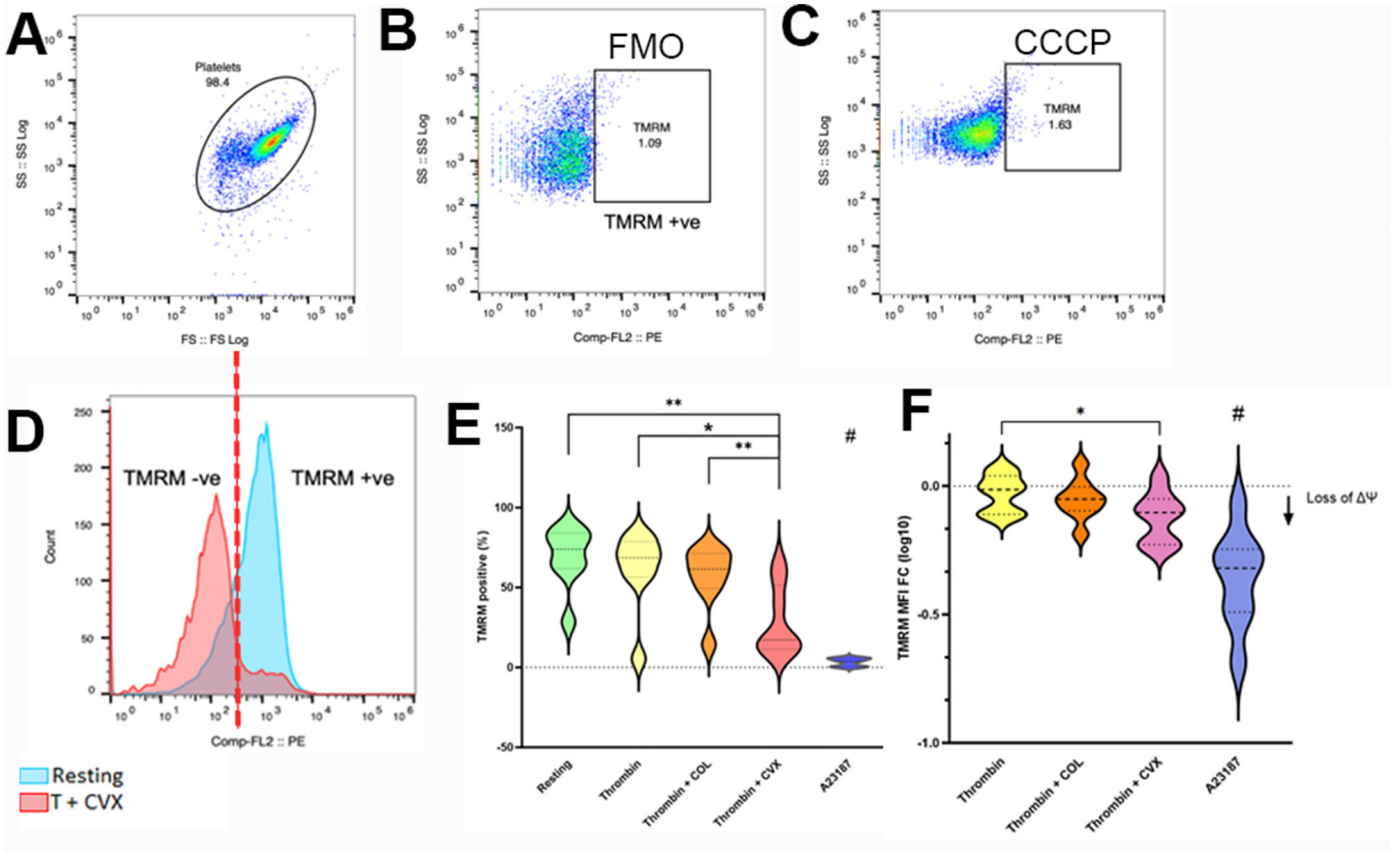
Flow cytometry analysis of mitochondrial membrane potential loss in response to various platelet agonists. Platelets isolated from 8 cats were unstimulated (resting) or activated with 0.001 U/ml thrombin with or without 4 μg/ml collagen (COL) or 100 ng/ml convulxin (CVX) after loading with tetramethylrhodamine methyl ester (TMRM). The extent of mitochondrial membrane potential loss was analyzed by flow cytometry and measured as percent (%) TMRM positive and fold change in median fluorescence intensity of TMRM (MFI FC) from resting platelets. **(A)** Platelets were identified using their forward (FS) and side scatter (SS) properties. **(B)** Fluorescent minus one (FMO) control, established in thrombin + COL-treated platelets, was used to identify TMRM positive platelets. **(C)** Basal expression of TMRM in platelets treated with carbonyl cyanide 3-chlorophenylhydrazone (CCCP) was used as negative control. **(D)** Histogram indicating the presence of intracellular TMRM in unstimulated platelets (blue) and loss of TMRM after thrombin + CVX stimulation (red). **(E)** Thrombin + CVX induced loss of mitochondrial membrane potential compared to all treatments, measured as percentage (%) cells. **(F)** Loss of mitochondrial membrane potential was measured as decreased fold change (log 10) in mean fluorescence intensity (MFI) of TMRM. Thrombin + CVX induced the most potent loss. A23187 served as negative control. *p < 0.05, **p < 0.01, # *p* < 0.05.

Platelets were standardized to 1 × 10^7^ cells/ml using Tyrode HEPES (pH 7.2, 5.5 mM dextrose, 1 mM CaCl_2_, no divalent cations) to a final volume of 200 μl in polypropylene culture tubes before loading with 320 nM tetramethylrhodamine methyl ester (TMRM) (Invitrogen, Eugene, OR) for 15 min at 37°C under gentle rocking. Tubes were protected from light and tube caps were secured in the aerobic position. TMRM-loaded platelets were then activated with 0.001 U/ml bovine alpha thrombin (5 min at 37°C, Haematologic Technologies, Inc., Essex Junction, VT) before treatment in the presence or absence of 4 μg/ml equine type I collagen (CHRONO-LOG Corp., Havertown, PA), or 100 ng/ml convulxin (Cayman chemicals, Ann Arbor, MI) for an additional 5 min. Unstimulated (resting) platelets served as positive control. Platelets treated with 20 μM carbonyl cyanide 3-chlorophenylhydrazone (CCCP) for 1 h (Invitrogen, Eugene, OR), and 10 μM A23187 for 10 min (Millipore Sigma, Burlington, MA) served as negative controls. An aliquot of platelets was then extracted, placed in flow cytometry tubes with prewarmed Tyrode HEPES (1:20 dilution) and analyzed using a 5- color flow cytometer (Beckman-Coulter FC500, Beckman-Coulter Inc., Miami, FL) without any fixation. Platelets were identified by forward- and side-scatter properties previously established using 0.9 μm and 3 μm calibration beads in the log scale as described and 10,000 events were analyzed for each experimental condition (Figure 2A) (6). In addition, the platelet gate was further validated using platelets labeled with a mouse anti-human monoclonal antibody conjugated to allophycocyanin against β3-integrin (CD61). Gating of TMRM-positive platelets were established using unstained and CCCP or A23187-treated platelets (Figures 2B, C). The number of TMRM-positive platelets, expressed as percent (%) positive, was compared among different agonist groups. To evaluate procoagulant tendency of platelets, the magnitude of ΔΨm was calculated as percent change using the following equation:


$$\begin{array}{l} \text{ΔΨm }\left(\text{\% change}\right)=\\ \;\frac{\left[\left(\text{\% TMRM activated}\right)-\left(\%\text{ TMRM resting}\right)\right]}{\left(\%\text{ TMRM resting}\right)}\times \;100 \end{array}$$

Median fluorescence intensity (MFI) was used to assess the drop in the fluorescence intensity of TMRM among different agonist groups. The response to agonists was compared to unstimulated or resting platelets from each cat and evaluated by the following equation:


$$\begin{array}{l} \text{TMRM MFI fold change }\left(\text{log}10\right)=\left({\text{log}}_{10}\text{ MFI Activated}\right)\\ \;-\;\left({\text{log}}_{10}\text{ MFI Resting}\right)\; \end{array}$$

Flow cytometry data were analyzed using commercially available software (FlowJo, Tree Str Inc., Ashland, OR) (Figure 1, Step 1).

#### 2) Quantification of phosphatidylserine externalization

**Figure 3:**
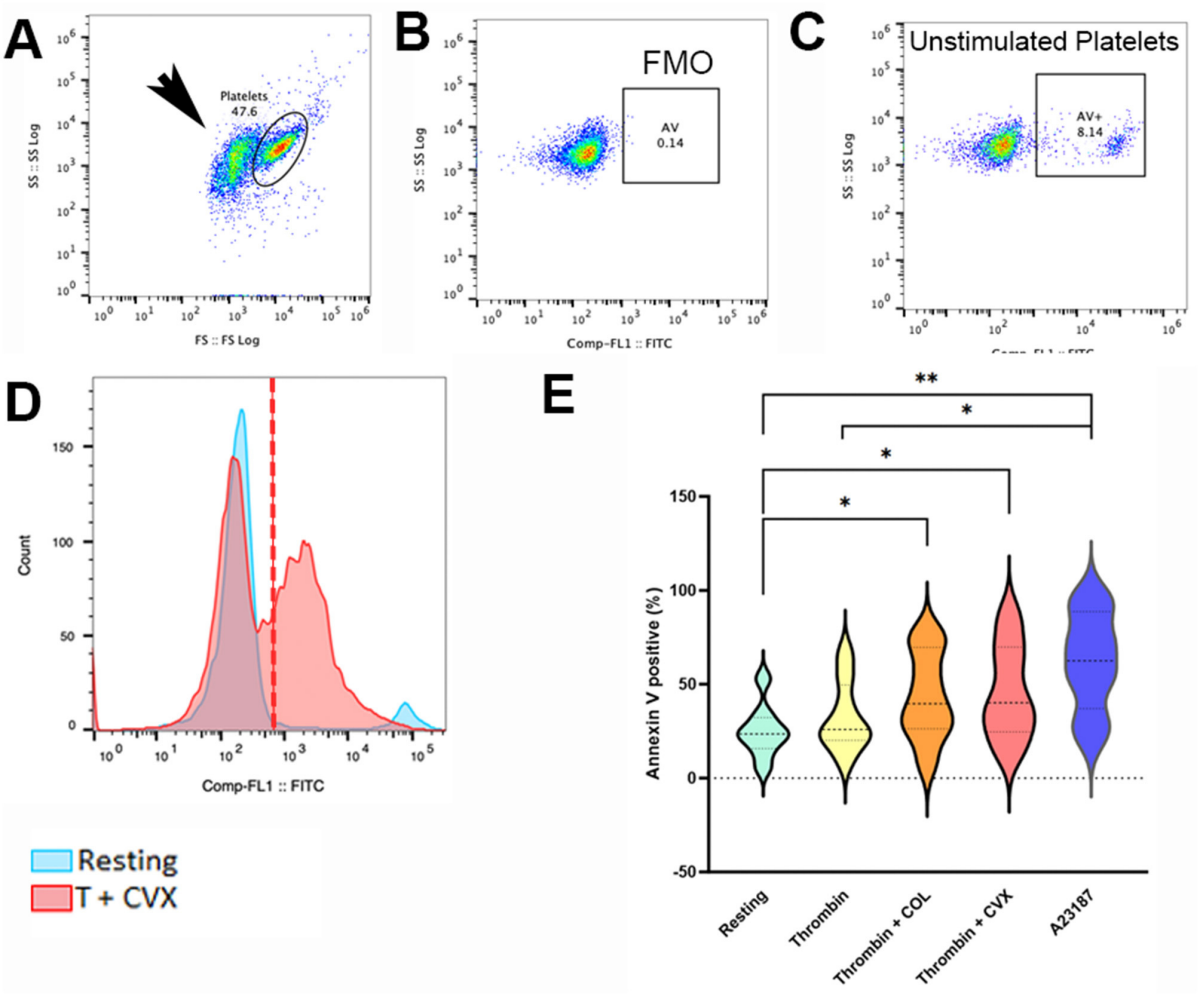
Flow cytometry analysis of phosphatidylserine externalization in response to various platelet agonists. Platelets isolated from eight cats were unstimulated (resting) or activated with thrombin with or without 4 μg/ml collagen (COL) or 100 ng/ml convulxin (CVX). Phosphatidylserine (PS) externalization was analyzed by flow cytometry and measured as percent (%) positive of annexin V (AV). **(A)** Platelets were identified by forward (FS) and side scatter (SS) properties. CD61 negative events, indicated by the arrow, were excluded from the main platelet gate. **(B)** Fluorescent minus one (FMO) control was established in thrombin stimulated platelets to identify AV-positive platelets. **(C)** Scatter dot plots demonstrating minimal PS externalization in unstimulated platelets. **(D)** After stimulation with thrombin + CVX (red), a substantial increase in AV positive platelets is shown in this representative histogram compared to unstimulated platelets (blue). **(E)** Thrombin with COL or CVX significantly increased PS externalization. A23187 served as the positive control. *p < 0.05. **p < 0.01.

The remaining platelets (100 μl) from step 1 were loaded with additional 1 mM CaCl_2_ for 15 min at 37°C (final concentration 2 mM). Platelets (Figure 3A) were then labeled with annexin V conjugated to fluorescein isothiocyanate (FITC) (1:200, Cat: 556419, BD Pharmingen, San Jose, CA) for 45 min at 37°C. Subsequently, samples were fixed in 0.1% methanol free paraformaldehyde (Thermo Scientific, Waltham, MA) for 30 min at room temperature. Resting platelets (Figures 3C, D) and A23187-treated platelets served as negative and positive controls, respectively. Fixed platelets were analyzed using flow cytometry as described above (Figure 1, Step 2). Annexin V-positive platelets were expressed as % positive platelets (Figures 3B, C). Procoagulant tendency was assessed as percent change based on unstimulated or resting platelets of each cat using the following equation:


$$\begin{array}{l} \text{Response to agonist}\left(\text{s}\right)\text{AV }(\%\text{ change})=\\ \;\frac{\left[\left(\%\text{ AV activated}\right)-\left(\%\text{AV resting}\right)\right]}{\left(\%\text{ AV resting}\right)}\times 100 \end{array}$$

#### 3) P-selectin expression assessment by flow cytometry

**Figure 4:**
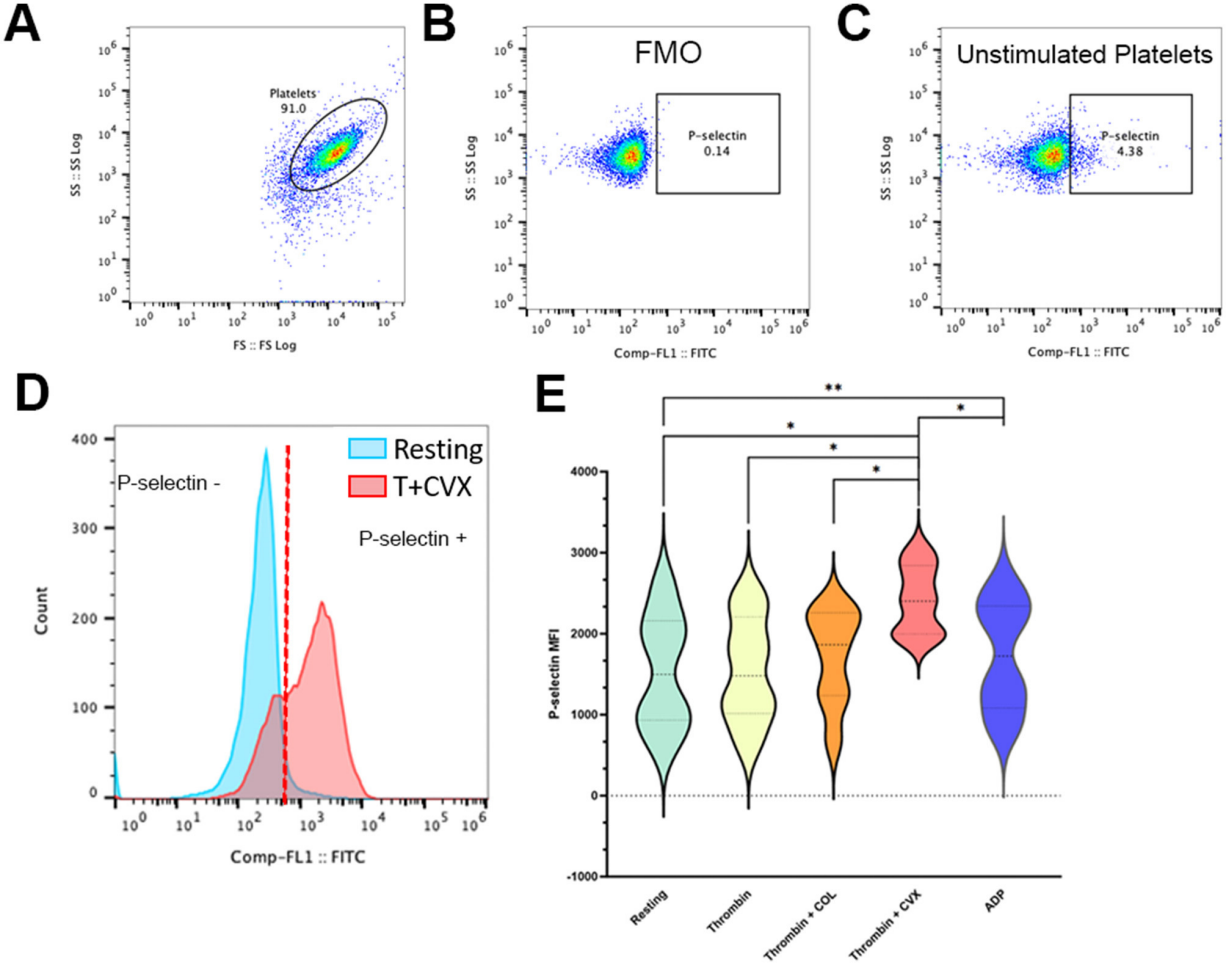
Presentative scatter dot plots and histograms demonstrating alpha granule release in feline platelets. Platelets from eight cats were either unstimulated (resting) or activated with 0.001 U/ml thrombin in the presence or absence of 4 μg/ml collagen (COL) and 100 ng/ml convulxin (CVX). Alpha granule release was analyzed by flow cytometry and measured as P-selectin median fluorescence intensity (MFI). **(A)** Representative scatter plots identifying platelets by their forward (FS) and side scatter (SS) properties. **(B)** Fluorescence minus one control (FMO), without P-selectin antibody, was used to establish P-selectin gate. **(C)** Basal expression of P-selectin is shown here in unstimulated platelets. **(D)** Representative histogram showing a platelet population before and after stimulation with thrombin and CVX. Note the shift in P-selectin negative platelets (blue) to P-selectin positive platelets after stimulation (red). **(E)** Thrombin and CVX induced a significant increase in alpha granule release. Platelets treated with ADP served as the positive control. *p < 0.05. **p < 0.005.

A separate aliquot of platelets, standardized to 1 × 10^7^ cells/ml with Tyrode HEPES (pH 7.2, 5 mM dextrose, no divalent cations) were prepared simultaneously with samples in steps 1 and 2. Platelets were stimulated as described in step 1. Unstimulated platelets (Figure 4C) and platelets treated with 20 μM adenosine diphosphate (ADP) (Sigma-Aldrich, St. Louis, MO) served as negative and positive controls, respectively. Following activation, platelets were labeled for 45 min at 37°C with rat anti-mouse monoclonal antibody conjugated to FITC specific to P-selectin (CD62p) (1:200, Clone: RB40.34, BD Biosciences, San Diego, CA) and a mouse anti-human monoclonal antibody conjugated to allophycocyanin against β3-integrin (CD61) (1:1000, Clone:VI-PL2, eBioscience, San Deigo, CA). Cells were fixed in 1% paraformaldehyde and analyzed by flow cytometry as described in step 1 (Figure 1, Step 3). Fluorescence minus one controls containing antibodies directed at either CD61 or CD62p were utilized to determine the positive events for P-selectin and integrin within that established platelet gate to a total of 10,000 events (Figures 4A, B). Compensation was achieved using mouse immunoglobulin G1 kappa isotype controls conjugated to FITC and APC (BD Biosciences, San Diego, CA). Surface P-selectin expression on platelets was quantified as median fluorescence intensity (MFI) compared among different agonist groups. To assess procoagulant tendency, response to agonists using unstimulated or resting platelets from each cat was evaluated by the following equation:


$$\begin{array}{l} \text{P }-\text{selectin MFI fold change }(\text{log}10)=\left({\text{log}}_{10}\text{MFI Activated}\right)\\ \;-\left({\text{log}}_{10}\text{ MFI Resting}\right)\; \end{array}$$

### Scoring system for determining the propensity of procoagulant platelet formation in cats

The propensity of procoagulant platelet formation of each subject was assessed by establishing a novel scoring system based on the magnitude of changes of the 3 aforementioned markers after *in vitro* treatments with the 3 agonist groups. A score of 1 for each marker was assigned to each cat if the magnitude of change exceeded the 40^th^ percentile of the data generated in the population. An individual with a total propensity score of 3 was considered to have the highest propensity for procoagulant platelet formation while a score of 0 was regarded as the lowest procoagulant tendency. For the purpose of this study, an individual was automatically assigned a score of 0 if the magnitude of P-selectin upregulation did not exceed the anticipated increase of >40^th^ percentile as apoptotic platelets do not undergo alpha granule release.

### Statistical analysis

Sample size was calculated based on previously established data with a minimum detectable effect of 30% in platelet activation and power of 80% with an alpha-priori of 0.05. A minimum of 6 cats was required; however, given the sensitivity of feline platelets with the anticipation of *in vitro* activation and clotting of blood samples, the sample size was increased to 8. D'Agostino and Pearson test and visual inspection of histograms were used to assess the normality of the data. Parametric data were represented as mean ± standard deviation and non-parametric data were presented as median and interquartile range (IQR). Normally distributed paired data were analyzed using *t*-tests while Wilcoxon signed-rank test was used to analyze non-parametric paired data. Treatment effect was analyzed by ANOVA of repeated measures or Kruskal-Wallis one-way analysis of variance with *post-hoc* analyses by Tukey test. Data were statistically significant with an alpha < 0.05. Fisher's exact test and construction of contingency tables were used to compare procoagulant propensity scores (categorical data) among the subjects. Data were analyzed using commercially available software (Prism 10.0, GraphPad Software, La Jolla, CA).

## Results

### Thrombin stimulation in the presence of convulxin resulted in a loss of mitochondrial membrane potential

A treatment effect on mitochondrial membrane potential (ΔΨm) was observed after platelets were treated with thrombin in the presence or absence of collagen (COL) or convulxin (CVX) (*p* = 0.0001). Compared to unstimulated (resting) platelets, ΔΨm, detected by a decrease in TMRM-positive platelets (%), was depolarized upon treatment with thrombin + CVX (resting = 73.95%, IQR 61.85–84.13 vs. thrombin + CVX = 17.15%, IQR 11.48–51.40; *p* = 0.0078) (Figure 2D). Of all agonists, thrombin + CVX was the most potent in reducing ΔΨm compared to thrombin (68.60%, IQR 56.53–78.70; *p* = 0.1953) and thrombin + COL (61.55%, IQR 49.43–71.28; *p* = 0.1094). A23187, a calcium ionophore, decreased ΔΨm significantly compared to resting, thrombin +/– COL/CVX (*p* = 0.0078) (Figure 2E). In addition, the loss of ΔΨm was measured as fold change (log 10) in mean fluorescence intensity (MFI) of TMRM. An overall decrease in TMRM intensity to agonists was recorded (*p* = 0.0005). Similar to percentage results, thrombin in the presence of CVX significantly reduced ΔΨm compared to thrombin (thrombin = −0.02 ± 0.07 vs. thrombin + CVX = −0.12 ± 0.09; *p* = 0.0376) (Figure 2F).

### Subsequent phosphatidylserine externalization was optimized with addition of calcium, collagen and convulxin after thrombin stimulation

Following the assessment of ΔΨm, externalization of PS was induced by additional loading of calcium and incubation with annexin V. A treatment effect on PS externalization was noted (*p* = 0.025). Thrombin (32.61% ± 18.90) alone was not effective at externalizing PS when compared to resting (resting = 25.16% ± 14.08; *p* = 0.11). When thrombin-activated platelets were stimulated further with COL or CVX, PS-positive platelets were markedly increased compared to resting platelets (thrombin + COL = 43.54% ± 23.79; *p* = 0.043; thrombin + CVX = 46.01% ± 25.60; *p* = 0.0172) (Figures 3D, E). A23187, which served as positive control, induced a significant increase in PS externalization (61.76% ± 26.37, *p* = 0.0079).

### Co-stimulation of platelets with thrombin and convulxin increased alpha granule secretion

A separate analysis of alpha-granule secretion was measured by P-selectin expression (MFI). An overall response to agonists in alpha-granule secretion was observed (*p* = 0.0063). We found that thrombin alone was not sufficient in inducing a significant increase in P-selectin when compared to resting platelets (resting = 1,542 ± 714.8 vs. thrombin = 1,553 ± 654.2; *p* = 0.9363). Similarly, treatment of platelets with collagen after thrombin stimulation induced a moderate increase in P-selectin but this increase was not statistically significant compared to resting platelets (thrombin + COL = 1,716 ± 593.2; *p* = 0.1925). Treatment of platelets with CVX after thrombin stimulation significantly increased alpha granule release (thrombin + CVX = 2,422 ± 420.2, *p* = 0.0128) (Figures 4D, E) and this increase was more profound that those observed in ADP-treated platelets (ADP = 1,736 ± 683.6; *p* = 0.0097) (Figure 4E).

### Development of a scoring system to assess procoagulant tendency of platelets

**Figure 5:**
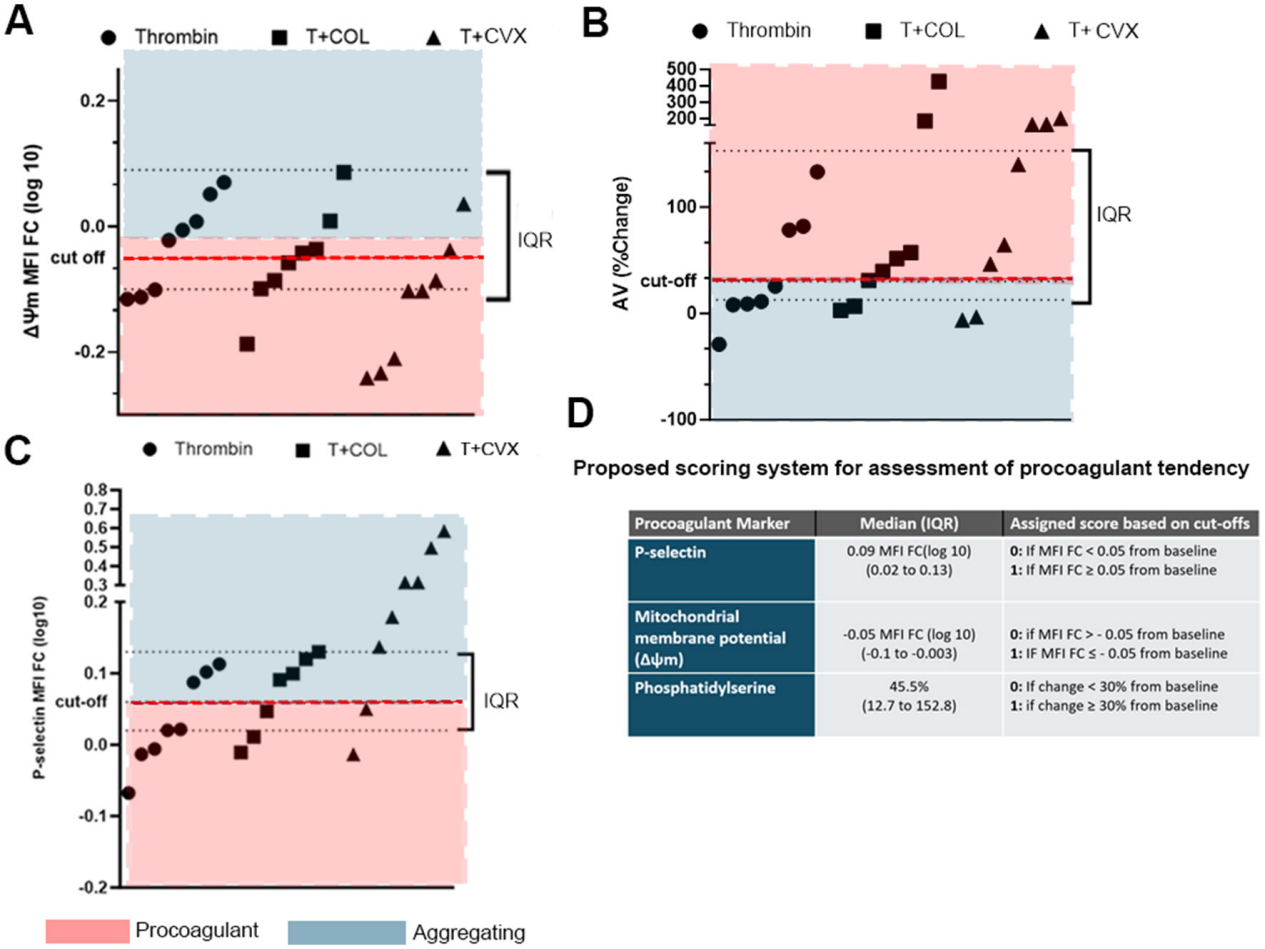
Distributions of platelet responses to thrombin in the presence or absence of collagen (COL) and convulxin (CVX). Arbitrary cut-offs, shown as red dotted lines, were established based on the medians and interquartile ranges of platelet responses to differentiate between procoagulant and activating platelets. Each point represents platelet response, measured as **(A)** loss of mitochondrial potential (Δψ, fold change in median fluorescence intensity), **(B)** Annexin V binding (% change) and **(C)** increase in P-selectin (fold change in median fluorescence intensity, MFI FC) in each cat. Interquartile ranges (IQR) are shown here as black dotted lines. **(D)** Table summarizing the scoring system and cut-off points used to establish procoagulant tendency.

A scoring system was established to evaluate the procoagulant tendency of platelets in individual cats. Given the low number of animals, the distribution of data was visually inspected and medians and IQRs were calculated for all 3 platelet markers among the agonist groups (thrombin, thrombin + COL, thrombin + CVX). As shown in Figure 5, arbitrary cut-offs were established based on the magnitude of changes in procoagulant markers induced by thrombin + COL to differentiate procoagulant from aggregating phenotypes. The following cutoffs, therefore, were established to determine if the measured procoagulant markers from an individual cat exceeded the procoagulant threshold: (a) increase in P-selectin expression with MFI FC_log10_ ≥ 0.05, (b) loss of ΔΨm with MFI FC_log10_ ≤ −0.05, and (c) ≥ 30% change in PS externalization following stimulation with agonist(s) (Figure 5). A score of 1 for each phenotype was assigned if the response to each agonist(s) exceeded the cut-off. A score of 0 for each procoagulant marker was assigned if the response was below the cut-off. A cat with a total score of 3 was considered to have the highest procoagulant tendency while an individual with a score of 0 was deemed to have the lowest procoagulant tendency. For the purpose of this study, individuals with a P-selectin score of 0 were automatically assigned a total score of 0 as their response to the specific agonists was considered apoptotic.

### Procoagulant tendency of platelets is increased the most in thrombin and convulxin treated platelets in individual cats

**Figure 6:**
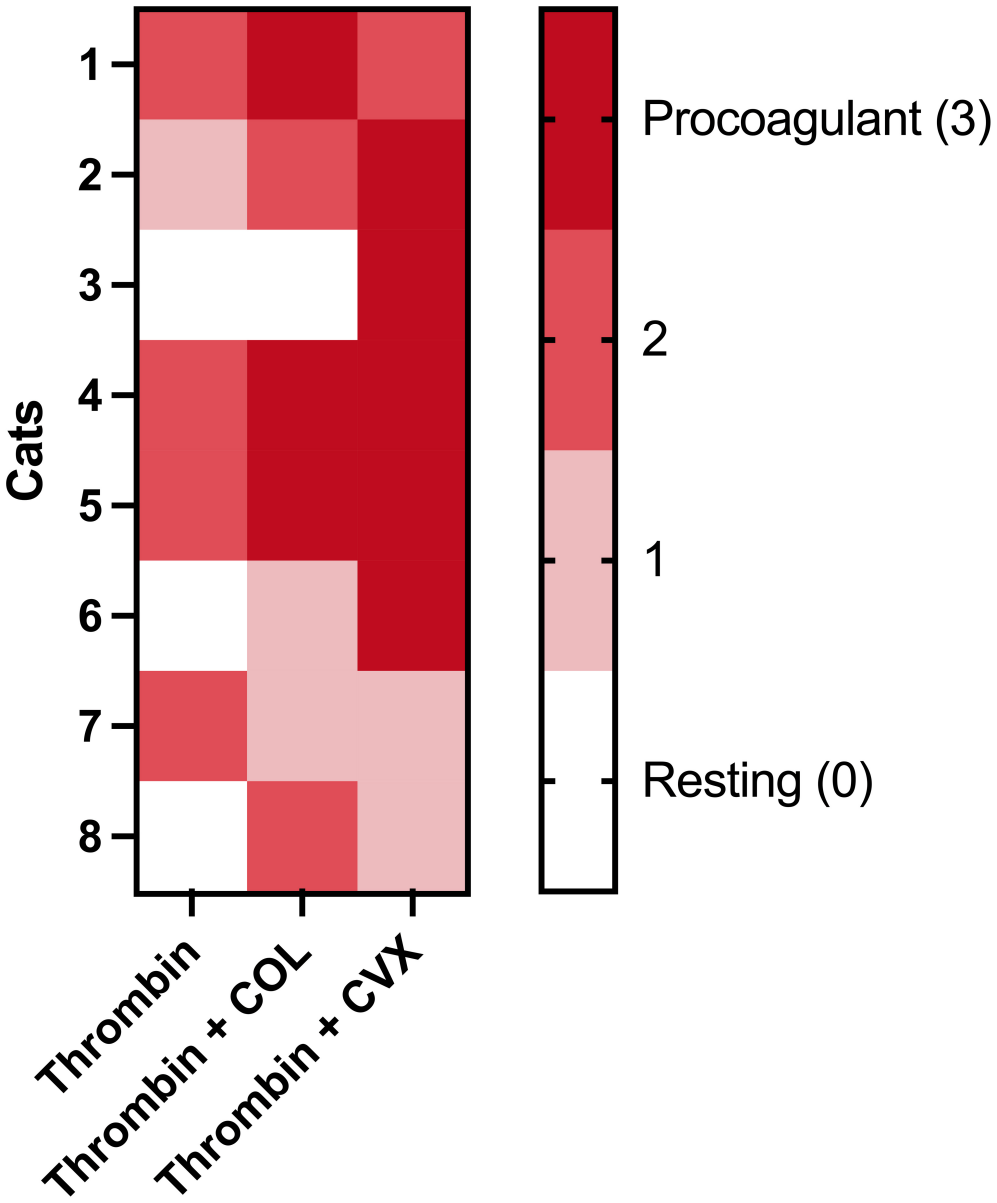
A heatmap showing the distribution of platelet procoagulant tendency scores in eight cats in various agonists. Platelets isolated from each cat were stimulated with thrombin, thrombin + COL or thrombin + CVX. Scores were assigned based on criteria according to procoagulant platelet markers after stimulation with agonists. A score of 3 represents high procoagulant platelet propensity in an individual while score of 0 represents resting or apoptotic platelet. The highest procoagulant tendency was noted upon thrombin + CVX stimulation.

Using the proposed scoring system, we evaluated the procoagulant tendency of the 3 agonist groups in each cat. Figure 6 summarizes the distribution of platelet procoagulant tendency scores among the 8 cats. As expected, the highest procoagulant tendency was noted upon platelet treatment with thrombin + CVX in which 6 of the 8 (75%) cats had a score of 2 and above. The number of cats with a procoagulant tendency score of 2 or 3 for thrombin and thrombin + COL treated platelets was 4/8 (50%) and 5/8 (62.5%), respectively. Two of the cats (number 3 and 6), had hyporesponsive platelets and thrombin in the presence or absence of COL was not strong enough to induce procoagulant phenotype until CVX was used as an agonist.

## Discussion

Here, we described a novel technique to characterize procoagulant platelet formation in cats using flow cytometry. Based on the magnitude of changes of procoagulant markers, we devised a novel scoring system to assess platelet procoagulant tendency in each individual cat. We demonstrated that of all the agonists, treatment of platelets with thrombin and CVX was the most effective at inducing alpha granule secretion and mitochondrial membrane depolarization. Thrombin in the presence of calcium, COL, or CVX induced PS externalization more effectively than thrombin alone. By combining the distribution of platelet response based on the three chosen markers, a novel scoring system was devised to evaluate the procoagulant tendency of platelets.

Although the role of procoagulant platelets in cats is unknown, thromboembolic disease remains a major contributor to morbidity and mortality in feline medicine (1, 2). A standardized technique to characterize procoagulant platelets in cats is, therefore, needed to not only identify cats at risk of thromboembolism but also to serve as therapeutic biomarkers. However, *ex vivo* studies on feline platelets and characterization of highly activated platelets like procoagulant platelets can be extremely challenging to conduct in clinical cats. A key feature of feline platelets is their increased propensity to activate in the absence of agonists, which is often characterized by irreversible shape change and high P-selectin expression. Platelets isolated from cats with conditions like cardiomyopathies, burn injuries and smoke inhalation are mostly positive for P-selectin (4–6). Once isolated, feline platelets are prone to undergo *in vitro* activation, which may be, in part, due to priming by endogenous epinephrine due to stress from animal restrain and increased platelet sensitivity to ambient temperature. Unlike species such as humans and pigs, feline platelets are cold sensitive with an activation threshold that begins around 30°C when membrane phospholipids undergo phase transition. This cold-induced phase separation causes leakage across the platelet membranes resulting in undesirable activation. Hence, the handling and processing of platelets at room temperature must be minimal to mitigate the impact of cold-induced activation (6, 22, 23). In addition, based on our experiences, feline platelets are susceptible to auto-aggregation upon treatment with ultra-low dose thrombin in the presence of calcium. A tailored approach is therefore needed to assess procoagulant platelet phenotypes and formation in cats.

To distinguish procoagulant platelets from other platelet subtypes in clinical patients, the International Society of Thrombosis and Haemostasias recommends a panel of 3 surface markers that can be detected by flow cytometry to differentiate between procoagulant and apoptotic platelets. These surface markers include P-selectin (CD62p), PS, and the platelet-specific receptor GPIX (CD42a) or α_IIb_ integrin (CD41, GPIIb) (21). It is important to recognize that while these guidelines serve to standardize the recognition of procoagulant platelets in humans, there are distinct species differences in platelet physiology that may pose some challenges when studying procoagulant platelets in cats. In human platelets, PAC-1 is a monoclonal antibody that recognizes the high-affinity conformation of integrin, α_IIb_β_3_ in activated platelets (21). During the process of procoagulant platelet formation, loss of ΔΨm facilitates the opening of the mitochondrial permeability transition pore complex (mPTP) creating supramaximal concentrations of calcium in the cytoplasm by favoring an efflux of calcium from the mitochondria. This sustained intracellular hypercalcemia activates calpain, a calcium-dependent protease, causing proteolysis of α_IIb_β_3_. The loss of α_IIb_β_3_ is a distinct feature of procoagulant platelets in humans and mice as they have lost the ability to aggregate (24, 25). For that reason, to distinguish procoagulant from aggregating platelets in humans, a decrease in PAC-1 binding in activated platelets is a hallmark of procoagulant phenotype (21). While P-selectin and PS can be measured on feline platelets by flow cytometry, there are no species-specific antibodies capable of detecting activated α_IIb_β_3_ integrin in cats. To overcome this, we utilized tetramethylrhodamine methyl ester (TMRM), a cell permeant cation, which is sequestered by intact mitochondria, to evaluate any changes in ΔΨm caused by mPTP opening in platelets. Thus, the loss of TMRM fluorescent signal served as a surrogate marker of mPTP opening, and potential cytoplasmic hypercalcemia and α_IIb_β_3_ hydrolysis. Given the temperature sensitive nature of feline platelets, it is critical to ensure that platelets are kept at 37°C with small variations in ambient temperature during TMRM loading. In addition, platelets must be maintained in an aerobic environment allowing the mitochondria to sustain proper functions of the electron transport chain.

Following mPTP opening, supramaximal calcium concentration in the cytoplasm stimulates the activation of TMEM16F, a scramblase enzyme, which facilitates the translocation of the negatively charged PS from the inner leaflet to the outer side of the plasma membrane. These negatively charged phospholipids serve as binding sites for the activation of coagulation zymogens to form tenase and prothrombinase complexes to amplify thrombin generation (24). Since aggregating platelets have minimal flipping of PS, it is one of the defined characteristics of procoagulant platelets. Detection of externalized PS via annexin V binding also requires supraphysiologic levels of extracellular calcium for adequate binding. Excessive calcium loading in a short period of time may lead to a rapid rise in cytosolic calcium caused by agonist-induced release of calcium stores within the dense tubular system. In addition, calcium loading may further exacerbate increased cytosolic calcium by calcium influx through channels such as ORAI-1 and TRPC6, which may force calcium entry to the mitochondria via the mitochondrial calcium uniporter (MCU), located on the inner mitochondrial membrane. This may artificially induce mPTP opening (25). We learned from our previous experiences of studying feline platelets that calcium loading with more than 1 mM CaCl_2_ at a time could led to shedding of microparticles, decrease in β3 integrin expression and formation of microaggregates in the absence of agonists (5). These observations indicate that feline platelets are sensitive to exogenous calcium and the presence of calcium could lead to premature activation, which may negatively affect our ability to characterize procoagulant markers on feline platelets. For that reason, we devised a stepwise approach to minimize *in vitro* activation. First, platelets were standardized with a buffer containing 1 mM CaCl_2_ before TMRM measurement in the presence or absence of platelet agonists. We found that this calcium loading strategy allows platelets to maintain ΔΨm in the absence of agonists and significant differences in ΔΨm could be detected once platelets were stimulated. Although the calcium concentration in our buffer was sub-physiologic, platelets from 2 cats were noted to have a loss of ΔΨm in the absence of platelet agonists. This may be secondary to high intra-assay variability as a result of *in vitro* activation or technical error during TMRM loading since resting platelets are expected to have the highest TMRM signal given their intact inner mitochondrial membrane. The variability in TMRM signal may have hindered our ability to detect significant differences between thrombin-stimulated and unstimulated platelets. Running duplicates or triplicates and calculating an average and co-efficient of variation of each treatment could potentially overcome the issue of assay variability.

After the measurement of ΔΨm, a cocktail of CaCl_2_ and fluorophore-conjugated annexin V was added to the remaining platelets for detection of PS flip. This small increment in calcium supplementation serves two purposes. First, not only does it avoid unnecessary *in vitro* activation, but it also mimics the cellular processes of sustained intracellular hypercalcemia that occur after mitochondrial membrane depolarization as mentioned above. Second, additional calcium is required since annexin V is a calcium-dependent phospholipid binding protein on PS. Since PS detection by annexin V cannot be performed in fixed cells due to membrane alterations, investigators are advised to trial different incubation times so that the shortest duration could be chosen to optimize PS detection in live platelets without inducing *in vitro* activation, apoptosis or necrosis due to prolonged exposure to agonists. Our incubation time of 45 min was determined by testing various durations based on our previous experiments and manufacturers' recommendation. Differences in flow cytometers can also influence the sensitivity of this detection. For this protocol, we tested 4 different incubation times (15, 30, 45, and 60 min), which showed that 45 min was the optimal duration for annexin V binding. Longer duration of incubation might result in platelet apoptosis. The addition of gly-pro-arg-pro could have also prevented clot formation as a result of longer incubation periods. Lastly, it is important to note that PS should be used in conjunction with other markers like P-selectin and ΔΨm loss to identify procoagulant platelets since platelets undergoing apoptosis also expose their PS.

The final marker that was utilized to characterize procoagulant response was P-selectin, which is a transmembrane protein specific to the platelet alpha granules. It is translocated to the plasma membrane upon degranulation with inside-out signaling and granule release (26). P-selectin is a reliable surface marker for detecting platelet activation and response to clopidogrel therapy in cats (6, 27). Of the 3 procoagulant markers, P-selectin plays an important role in distinguishing procoagulant from apoptotic platelets. It is still unclear if procoagulant platelets are the outcome of necrosis hence both cellular pathways may result in similar phenotypes. Like procoagulant platelet formation, apoptosis also causes inner mitochondrial membrane depolarization and ΔΨm loss. However, during apoptosis, this is mediated by intrinsic or extrinsic stimuli, which lead to caspase 8 activation and cytochrome C release from the mitochondria. The assembly of apoptosomes and caspase 3 cleaves platelet scramblase causing PS to be externalized. The distinguishing characteristic between these two populations of platelets is upregulation of surface P-selectin (28–30). While procoagulant platelets express P-selectin on the outer membrane in response to strong agonists, apoptotic platelets do not undergo alpha granule release. This was shown in two studies in which investigators treated human and canine platelets with ABT-737, an inducer of apoptosis, and found minimal P-selectin expression (29, 30). However, it is unknown if platelets treated with a combination of thrombin and COL or CVX could induce apoptosis as shown by a lack of P-selectin upregulation. For this reason, we categorized platelets from individuals with low P-selectin upregulation to have platelet apoptotic tendency in our scoring system regardless of the degree of ΔΨm loss and PS flip.

The incubation period of 45 min of P-selectin antibody followed by fixation was based on our extensive experiences evaluating platelet activation in clinical cats using flow cytometry (4–6, 11). Due to the temperature-sensitive nature of feline platelets, we found that immunolabeling at an ambient temperature of 37°C optimizes P-selectin detection in feline platelets. It is important to note that platelets could be falsely positive for P-selectin if the fixative is too concentrated or if the duration of fixation is too long. Unstimulated platelets in cats are often positive for P-selectin after isolation due to their sensitive nature to temperature changes and shear stress caused by centrifugation. We, therefore, recommend intermittent resting periods of up to 30 min after blood draw and centrifugation. In addition to the handling and processing of platelets, the temperament of each cat and the quality of venipuncture can affect the quality of platelets prior to experimental manipulation. With careful sample handling and processing, our data showed that 6 of the 8 cats had relatively well rested platelets with < 30% of platelets being positive for P-selectin. Two of the cats had P-selectin levels >50% after isolation, which likely limited our ability to detect the true magnitude of alpha granule secretion in those individuals.

Low dose thrombin of 0.001 U/ml was chosen in this study to limit *in vitro* aggregation that is often observed with treatments of platelets with thrombin and calcium. *In vitro* aggregation can interfere with antibody binding, detection of procoagulant markers and obstruct the flow cytometer fluidic system. Based on our previous experiences of studying feline platelets, high dose thrombin at 0.1 U/ml elicits a maximum effect on aggregation and activation while a dose of 0.05 U/ml induces robust P-selectin expression without complete aggregation on light transmission aggregometry (5, 6, 12). Given that cats with HCM have activated platelets, low dose thrombin also mimics *in vivo* hypercoagulability. Moreover, COL or CVX in addition to thrombin could lead to instantaneous aggregation given the sensitive nature of feline platelets. Alternatively, investigators can use gamma-thrombin instead of alpha-thrombin due to its reduced activity toward fibrinogen and thrombus formation. However, its potency in feline platelet platelets is not well understood. The concentration of COL (4 ug/ml) was selected based on our preliminary testing using whole blood electrode impedance aggregometry (data not shown). We found that the recommended COL concentration by the manufacturer elicited an aggregation response that is about two times higher than that in human platelets; thus, this concentration of COL was considered optimal for inducing procoagulant platelets. Our laboratory has previously shown that 2.5 μM to 5 μM A23187 could not sufficiently induce PS flip and budding of platelet-derived microvesicles in feline platelets (5). Similar to multiple human publications, we find that 10 μM A23187 was effective at inducing procoagulant platelets and, therefore, was utilized as positive controls (24, 30, 31). The dose of 100 ng/ml CVX was adopted from multiple human studies on procoagulant platelets and is shown to elicit a full response consistently (31, 32). However, given the species differences in platelet response to agonists, a dose response of CVX is needed to establish an optimal dose for studying procoagulant platelets in clinical cats.

In agreement with human data, our study showed that stimulation of protease activating receptor via thrombin fails to induce procoagulant phenotypes without the synergistic activation of GPVI in feline platelets. The addition of GPVI agonists, like COL and CVX, to thrombin resulted in the most pronounced procoagulant phenotypes (19, 24, 30, 31, 33). We established cut-offs for our scoring system based on the changes in markers in thrombin and COL activation due to the following reasons: First, thrombin alone was inadequate in inducing procoagulant phenotypes since most cats (P-selectin 5/8, AV 5/8, ΔΨm 4/8) fell within or outside the lower quartile of those activated by thrombin and COL. Second, co-stimulation of platelets with thrombin and CVX resulted in a majority of cats within or outside the higher quartile range when compared to the 2 agonist groups. These observations indicate that while CVX has a similar signal transduction pathway to COL via GPVI, there are some key differences between these 2 agonists. For instance, COL activates platelets via GPVI and α_2_β_1_ integrin. Also, CVX, a snake venom toxin, binds to platelet GPVI with high affinity to elicit tyrosine kinase phosphorylation leading to a more robust signaling cascade compared to COL (34). Given the potency of CVX to induce procoagulant phenotypes with thrombin, the authors believe that this combination of agonists may limit our ability to assess platelet heterogeneity in clinical cats. A combination of thrombin and COL may, therefore, be more sensitive to detect procoagulant tendency as they induce more physiologic responses than thrombin and CVX.

Instead of characterizing and quantifying procoagulant platelets by assessing all 3 surface markers simultaneously, we developed a scoring system based on the collective data from each of the 3 markers to determine the procoagulant tendency of platelets in individual cats. Quantifying procoagulant platelets in cats can be difficult due to the sensitive nature of feline platelets as mentioned above. A stepwise approach characterizing each procoagulant phenotype allows for a more physiologic assessment. This method, however, has considerable limitations. First, separate analyses of PS and P-selectin on platelets might have limited our ability to separate apoptotic from procoagulant platelets since PS are exposed during apoptosis. However, during the process of designing this protocol, attempts of simultaneous detecting PS and P-selectin not only resulted in suboptimal P-selectin antibody binding but also *in vitro* clot formation. Alternatively, investigators can utilize washed platelets, or dilute platelets in standardized concentrations of plasma to minimize fibrinogen interference of antibody binding. Future studies should investigate the feasibility of co-detecting P-selectin or other activation markers such as CD40L and PS in differentiating apoptotic from procoagulant platelets in cats to mitigate this limitation.

Second, the workflow established can be technically challenging given the time-sensitive nature of ΔΨm and PS analyses in live platelets, which could limit its utility in clinical settings. We recommend that the sequential detection of TMRM and PS to be done within 3 h after isolation to avoid platelet apoptosis. Due to the potential of *in vitro* clot formation, an alternative approach using washed or gel-filtered platelets could be adopted. Resuspension of platelets with a buffer containing a standard amount of autologous or pooled feline plasma could also allow simultaneous detection of fibrinogen binding. Fibrinogen binding experiments could be carried out to verify a decrease in binding to α_IIb_β_3_ integrin in the procoagulant platelets such as fibrinogen coated microfluid channel or assessing the lysis/internalization of integrin using Western blots. In contrast to studies in human platelets, which induce procoagulant platelets by simultaneous treatment of platelets with thrombin and a GPVI agonist like COL or CVX, we activated platelets with COL or CVX after a brief duration of thrombin stimulation. While this deviates from standard practice, priming of platelets by low-dose thrombin before the addition of COL or CVX could prevent instantaneous aggregation and fibrin formation in the presence of calcium. Further studies are needed to determine if the variation in platelet treatment could yield different results in feline platelets. Lastly, the total number of procoagulant platelets was not quantified, which may further limit our ability to characterize the dichotomous pattern of procoagulant and aggregating platelets in clinical patients. With various flow cytometry devices available, we recommend investigators to test the optimal concentrations and incubation period for annexin V binding enhancing the sensitivity of the assay and preventing undesirable *in vitro* activation.

Overall, this study provides a framework for future bench-top and clinical studies as it is a step toward standardizing procoagulant platelet phenotyping and propensity scoring in cats. Since current risk factors rely mainly on echocardiographic findings, procoagulant tendency of platelets in cats may be a potential biomarker to predict CATE. Further clinical studies are needed to validate our scoring system in cats with cardiomyopathies and to identify pathological thresholds in clinical cats. Also, given the variable response to antiplatelet drugs in cats, assessing procoagulant platelets in relation to clopidogrel resistance in cats may offer new antithrombotic targets to optimize the prevention and treatment of CATE.

## Conclusion

This study provides the first step of devising a reliable and sensitive diagnostic tool to identify procoagulant platelets and characterize their phenotypes to study their clinical relevance in cats with HCM.

## Data Availability

The raw data supporting the conclusions of this article will be made available by the authors, without undue reservation.
